# A case for resonant X-ray Bragg diffraction by a collinear antiferromagnet Li_2_Ni_3_P_4_O_14_

**DOI:** 10.1107/S2052520625009485

**Published:** 2025-11-13

**Authors:** Stephen W. Lovesey

**Affiliations:** a ISIS Facility STFC Didcot OxfordshireOX11 0QX United Kingdom; b Diamond Light Source Harwell Science and Innovation Campus Didcot OxfordshireOX11 0DE United Kingdom; c Department of Physics Oxford University Oxford OX1 3PU United Kingdom; Politecnico di Milano, Italy

**Keywords:** antiferromagnet, resonant X-ray scattering, Bragg diffraction, anapoles, chiral signature

## Abstract

Magnetic axial and polar (Dirac) nickel multipoles contribute to resonant X-ray Bragg amplitudes in a symmetry-informed analysis of monoclinic Li_2_Ni_3_P_4_O_14_ presented for future diffraction experiments. Magnetic long-range order below a temperature of ≃ 14.5 K can be viewed as a two-dimensional trimerized antiferromagnet with Ni ions in two Wyckoff positions in the centrosymmetric (1) magnetic space group *P*2_1_/*c*. It permits the coupling to circular polarization in the primary X-ray beam, unlike the corresponding diffraction by an antiferromagnet characterized by anti-inversion (1′) and a linear magnetoelectric effect, *e.g.* historically significant chromium sesquioxide (Cr_2_O_3_) and Cu_2_(MoO_4_)(SeO_3_) [Lovesey & van der Laan (2024 ▸). *Phys. Rev. B***110**, 174442]. The space group is inferred from neutron Bragg diffraction patterns, without an allowance for permitted Dirac dipoles (anapoles) and quadrupoles [Chikara *et al.* (2025). *Phys. Rev. B***112**, 014438].

## Abstract 2

Amplitudes for resonant X-ray diffraction by collinear antiferromagnet Li_2_Ni_3_P_4_O_14_ that include permitted Dirac multipoles, *e.g.* anapoles, are investigated. Chiral signatures for the rotation of X-ray helicity are described.

## 1. Introduction

Electronic correlations in conjunction with spin-orbit coupling in solids create valuable and enigmatic effects (Pourovskii *et al.*, 2025 ▸). These include the magnetoelectric effect, collinear antiferromagnetic order, and so-called hidden orders in which a state of matter exists without an easily detectable order parameter. Scattering techniques often have much to offer in gathering incisive information on properties of solids not available with other experimental methods. For example, Néel (1932 ▸) proposed antiferromagnetism and Shull & Smart (1949 ▸) recognized that magnetic neutron Bragg diffraction could provide the concrete evidence. To this end, diffraction patterns were collected from powdered MnO that presents magnetic order below a temperature of ≃122 K. A difference in patterns from samples at room temperature and at ≃80 K revealed strong magnetic Bragg spots at positions not allowed on the basis of the face-centred cubic unit cell of MnO.

A meaningful analysis of a Bragg diffraction pattern requires appreciation of the symmetry properties of the radiation-matter interaction and those of the illuminated sample (Collins *et al.*, 2007 ▸; Collins & Bombardi, 2010 ▸; Lovesey & Balcar, 2013 ▸; Winkler & Zülicke, 2025 ▸). For the sample, an extended Neumann’s principle (Neumann, 1885 ▸; Cracknell, 1975 ▸) imposes space-inversion symmetry and time-inversion symmetry for a resonant ion. Translations absent in the magnetic crystal class appear in the magnetic space group {Bilbao Crystallographic Server (https://www.cryst.ehu.es) Belov–Neronova–Smirnova [BNS] setting of magnetic space groups}. Discrete symmetries of the radiation-matter inter­action are spatial-inversion symmetry, time-inversion symmetry and photon polarization. Crystals of berlinite (*K*-absorption edge of Al), tellurium (*L*_1_ edge) and quartz (*K* edge of Si) form in space groups *P*3_1_21 (No. 152 right-handed screw) or *P*3_2_21 (No. 154 left-handed screw), and absolute chirality is verified by Bragg diffraction of circularly polarized X-rays tuned in energy to the specified absorption edges (Tanaka *et al.*, 2010 ▸). The most probable absorption event is the parity-even (axial) electric dipole–electric dipole (*E*1–*E*2) with atomic transitions 1*s* → 3*p* for Al and Si, and 2*s* → 5*p* for Te. The parity-odd (polar) electric dipole–electric quadrupole (*E*1–*E*2) absorption event plays a minor role. Parity-even *E*1–*E*2 and *E*2–*E*2 absorption events include nonmagnetic (time-inversion symmetry even) and magnetic (time-inversion symmetry odd) processes. The latter can reveal the long-range magnetic order of familiar axial dipole moments. The corresponding dipoles in a parity-odd *E*1–*E*2 absorption event are less familiar anapoles, also known as toroidal moments. Magnetic monopoles, which have not been observed (Milton, 2006 ▸), and anapoles possess the same discrete symmetries, namely, parity-odd and time-odd. These symmetries are manifested in a spin anapole (**n** × **S**) created with the vector product of position **n** and spin **S** of an electron.

**Figure 1:**
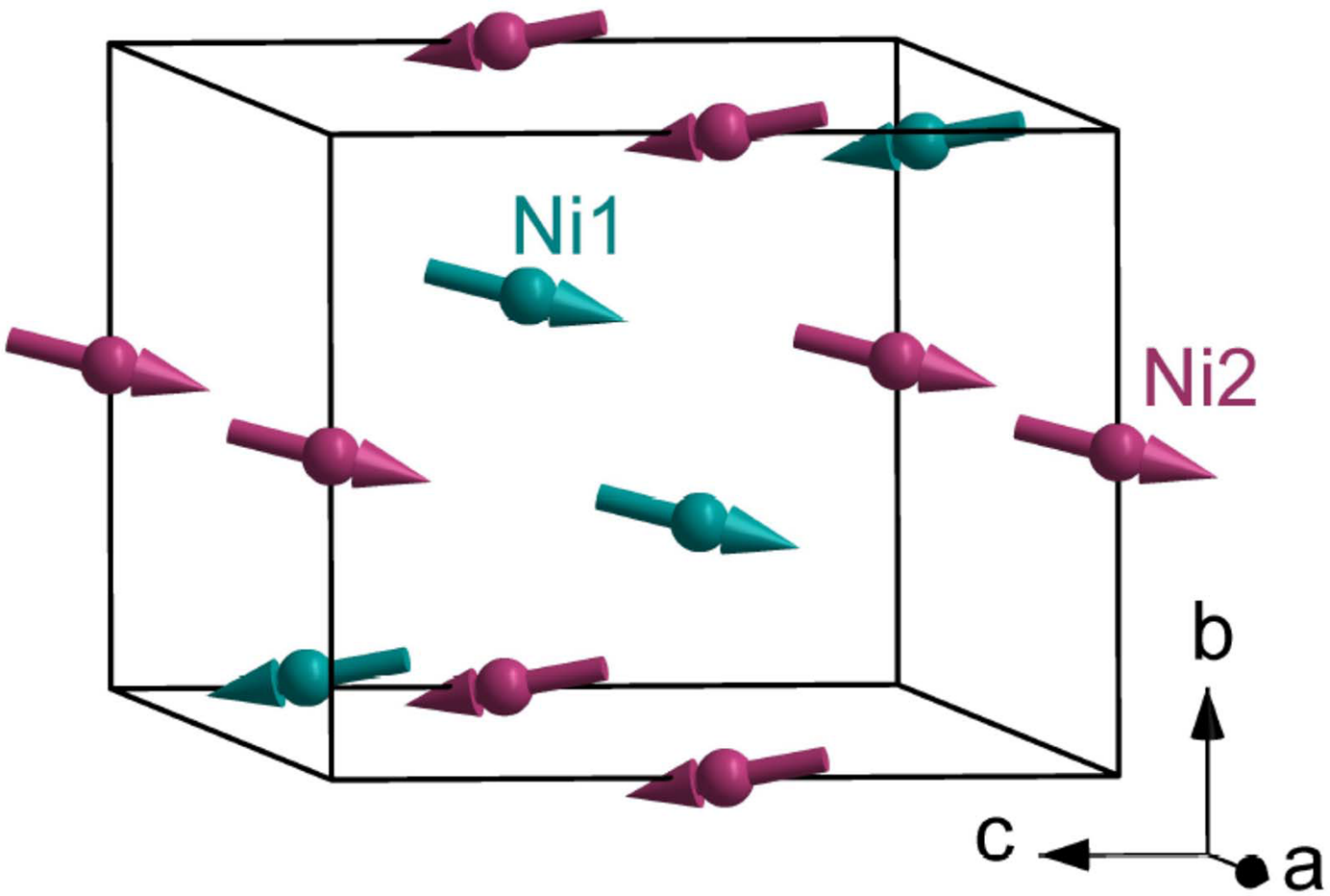
Axial dipoles in Li_2_Ni_3_P_4_O_14_ using magnetic symmetry *P*2_1_/*c* (No. 14.75, propagation vector **k** = 0, Bilbao [BNS]). They are in Wyckoff general positions 4*e* for Ni1 (blue arrows) and 2*c* for Ni2 (red). Chikara *et al.* (2025 ▸) constrained the two sites to have identical dipole moments and the inferred values are used in the figure.

A magnetic ground state for Li_2_Ni_3_P_4_O_14_ depicted in Fig. 1 ▸ has been proposed on the basis of magnetic susceptibility and specific heat measurements, neutron powder diffraction and neutron polarization analysis (Chikara *et al.*, 2025 ▸). Magnetic long-range order below a temperature of ≃ 14.5 K can be viewed as a two-dimensional trimerized antiferromagnet involving two independent Ni ions. The monoclinic magnetic space group assigned by the authors possesses a propagation vector = (0, 0, 0), commensurate collinear antiferromagnetic order and a ferromagnetic component parallel to the unique crystal axis, *b*. The permitted coupling of the magnetic order to photon circular polarization in the primary beam is one delineated characteristic of symmetry-informed amplitudes for resonant X-ray Bragg diffraction. Furthermore, Wyckoff positions in Fig. 1 ▸ differ with respect to spatial-inversion symmetry. Polar magnetic multipoles are permitted at the non-centrosymmetric position, including anapoles visible in resonant X-ray and neutron Bragg diffraction patterns (Fernández-Rodríguez *et al.*, 2010 ▸; Lovesey *et al.*, 2019 ▸).

Resonant X-ray Bragg diffraction by magnetic V_2_O_3_ illustrates the puissance of the technique (Paolasini *et al.*, 1999 ▸; Paolasini *et al.*, 2001 ▸; Lovesey & Knight, 2000 ▸). Bragg spots forbidden in the diffraction pattern of the monoclinic structure are due to orbital magnetism and not orbital order as originally proposed. The experiment exploited a relatively weak *E*2 absorption event at the vanadium *K* edge. Turning to a second example and a high-*T*_c_ material, emergence of the pseudo-gap phase with time-reversal violation in underdoped HgBa_2_CuO_4+δ_ (Hg1201) diminishes Cu site symmetry. Specifically, the Cu centre of inversion symmetry is lost, and Dirac multipoles herald the onset of the enigmatic phase (Lovesey & Khalyavin, 2015 ▸). Analysis of magnetic neutron diffraction patterns for Hg1201 yield the ortho­rhombic magnetic symmetry of the pseudo-gap phase (Bourges *et al.*, 2021 ▸; Croft *et al.*, 2017 ▸; Bourges *et al.*, 2018 ▸; Fechner *et al.*, 2016 ▸).

## 2. Magnetic structure

The magnetic structure adopted for Li_2_Ni_3_P_4_O_14_ (*P*2_1_/*c*, No. 14.75, Bilbao [BNS]) is depicted in Fig. 1 ▸ with nickel ions (Ni^2+^, 3*d*^8^) in Wyckoff general positions 4*e* and special positions 2*c* (Chikara *et al.*, 2025 ▸). Positions 4*e* are devoid of any symmetry and 2*c* are centrosymmetric. Nickel ions in positions 2*c* are permitted axial (parity even) multipoles alone, while positions 4*e* are permitted axial and Dirac multipoles. Reflection conditions include (0, 2*n*, 0) and (0, 0, 2*n*), and the monoclinic cell possesses a unique axis *b* Bilbao [BNS]. Magnetic multipoles are specified in orthogonal axes labelled (ξ, η, ζ) derived in standard form, namely, ξ ∝ a*, η ∝ **b**, ζ ∝ **c**, with reciprocal lattice vectors a*∝ *a*(1, 0, 0), b* ∝ *b*(0, 1, 0), c* ∝ *c*[−cos(β_o_), 0, sin(β_o_)] and an obtuse angle β_o_ ≃ 110.31° (Chikara *et al.*, 2025 ▸).

Turning to the actual calculation of a scattering amplitude using an atomic wavefunction, the practice of replacing matrix elements by those of convenient operators with strong physical appeal has a long history. In condensed matter physics, it is perhaps best known through use of operator equivalents in electron paramagnetic resonance by Elliott and Stevens (Abragam & Bleaney, 1970 ▸). Likewise, electronic multipoles, created with irreducible spherical tensors, to represent time-even (charge like) and time-odd (magnetic) quantities are widespread in modern physics. Specifically, matrix elements of the spin anapole (**n** × **S**) mentioned in the *Introduction*[Sec sec1] can represent a contribution to the scattering amplitude from Ni ions in Wyckoff positions 4*e*.

In our adopted description of electronic degrees of freedom, Ni ions are assigned spherical multipoles 〈*O*^*K*^_*Q*_〉 of integer rank *K* with projections *Q* in the interval −*K* ≤ *Q* ≤ *K* (Lovesey *et al.*, 2005 ▸; Lovesey & Balcar, 2013 ▸). Angular brackets denote the time-average, or expectation, value of the enclosed spherical tensor operator. Cartesian and spherical components *Q* = 0, ±1 of a vector **n** = (ξ, η, ζ) are related by ξ = (*n*_−1_ − *n*_+1_)/√2, η = *i*(*n*_−1_ + *n*_+1_)/√2, ζ = *n*_0_. As one example, the spin anapole (*K* = 1) is proportional to the multipole 〈(**n** × **S**)_*Q*_〉. A complex conjugate of a multipole is defined as 〈*O*^*K*^_*Q*_〉* = (−1)^*Q*^ 〈*O*^*K*^_−*Q*_〉, meaning the diagonal multipole 〈*O*^*K*^_0_〉 is purely real. The phase convention for real and imaginary parts labelled by single and double primes is 〈*O*^*K*^_*Q*_〉 = [〈*O*^*K*^_*Q*_〉′ + *i*〈*O*^*K*^_*Q*_〉′′]. Whereupon Cartesian dipoles are 〈*O*^1^_ξ_〉 = −√2 〈*O*^1^_+1_〉′ and 〈*O*^1^_η_〉 = −√2 〈*O*^1^_+1_〉′′.

A structure factor (Lovesey *et al.*, 2005 ▸)
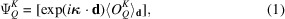
delineates the Bragg diffraction pattern for a reflection vector **κ** defined by integer Miller indices (*h*, *k*, *l*). The sum is over positions **d** used by Ni ions in *P*2_1_/*c* (No. 14.75, magnetic crystal class 2/*m*). Reflection conditions are derived from Ψ^*K*^_*Q*_ by considering its value for even *K* and *Q* = 0, and a parity-even signature σ_π_ = +1 (the structure factor for nuclear scattering). Bulk magnetic properties are defined by Ψ^*K*^_*Q*_ evaluated for *K* = 1 (dipole), σ_π_ = +1 and *h* = *k* = *l* = 0.

In more detail, equation (1) requires information about the relevant Wyckoff positions found in the BSC table MWYCKPOS for the magnetic symmetry of interest. Wyckoff positions are related by operations listed in the table MGENPOS (Bilbao [BNS]). Taken together, the two tables provide all information required to evaluate equation (1) and, thereafter, all X-ray diffraction amplitudes.

Henceforth, axial (σ_π_ = +1) and polar (σ_π_ = −1) multipoles are denoted by 〈*T*^*K*^_*Q*_〉 and 〈*G*^*K*^_*Q*_〉, respectively. The time signature σ_θ_ of 〈*T*^*K*^_*Q*_〉 is σ_θ_ = (−1)^*K*^, *i.e.* even *K* axial multipoles are non-magnetic. Dirac multipoles 〈*G*^*K*^_*Q*_〉 are magnetic with σ_θ_ = (−1) for all *K*. We find
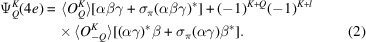
Spatial phase factors in equation (2) are α = exp(*i*2π*hx*), β = exp(*i*2π*ky*) and γ = exp(*i*2π*lz*). Estimates of the general coordinates (*x*, *y*, *z*) for Ni ions are given by Chikara *et al.* (2025 ▸). Wyckoff positions 2*c* are centres of inversion symmetry (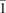, σ_π_ = +1) and nothing more. The result
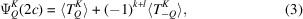
complies with a reflection condition (*k* + *l*) = 2*n*.

## 3. X-ray diffraction

**Figure 2:**
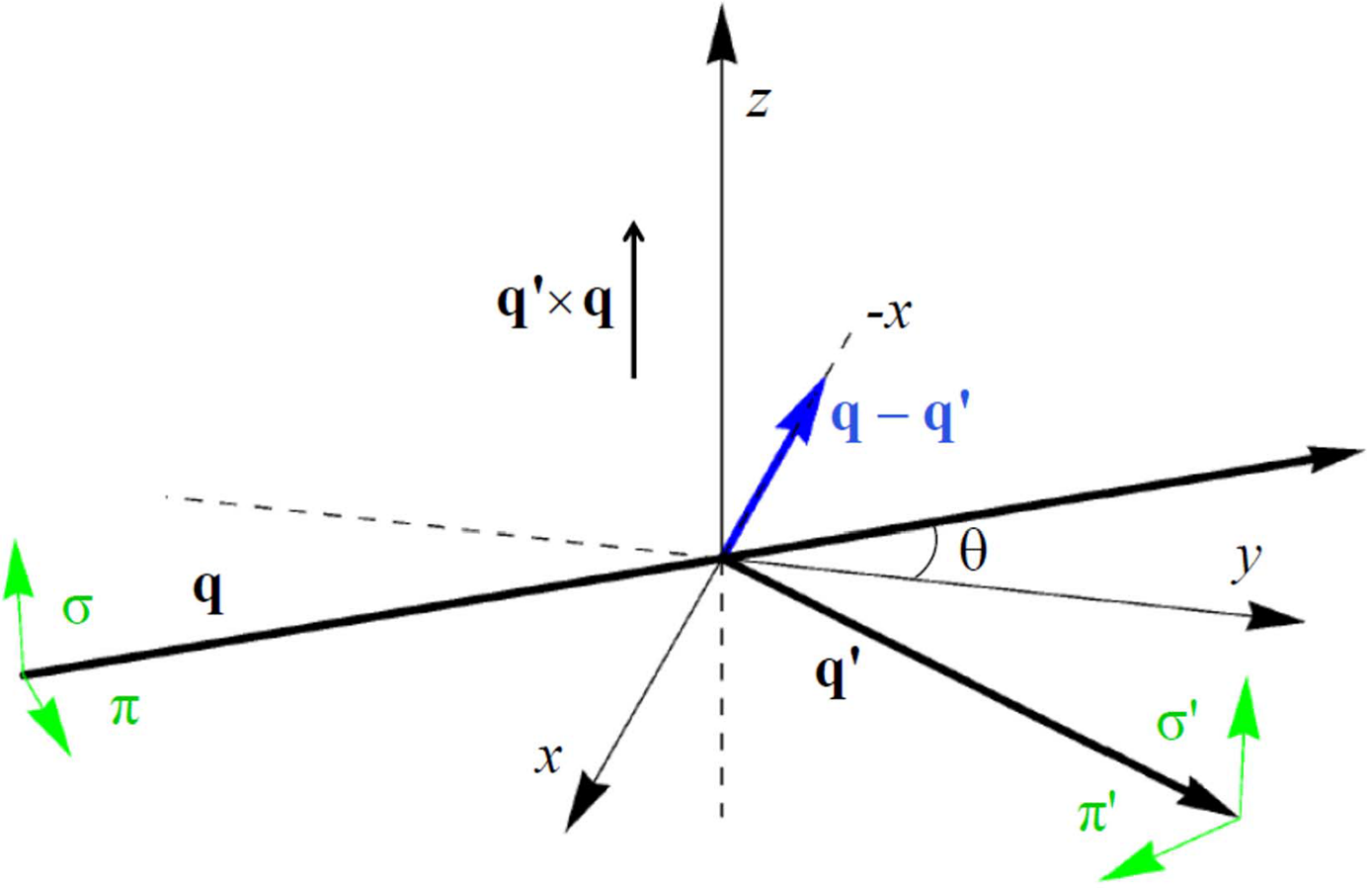
Primary (σ, π) and secondary (σ′, π′) states of polarization. Corresponding wavevectors **q** and **q**′ subtend an angle 2θ. The Laue condition for diffraction is met when **q** − **q**′ coincides with a reflection vector (*h*, *k*, *l*) of the monoclinic reciprocal lattice. Crystal vectors that define local axes for Ni ions (ξ, η, ζ) and the depicted Cartesian (*x*, *y*, *z*) coincide in the nominal setting of the crystal.

Tuning the energy of the X-rays to an atomic resonance has two obvious benefits in diffraction experiments (Paolasini, 2014 ▸). In the first place, there is a welcome enhancement of Bragg spot intensities and, secondly, spots are element specific. States of X-ray polarization, Bragg angle θ, and the plane of scattering are shown in Fig. 2 ▸. A conventional labelling of linear photon polarization states places σ = (0, 0, 1) and π = [cos(θ), sin(θ), 0] perpendicular and parallel to the plane of scattering, respectively. Secondary states σ′ = σ and π′ = [cos(θ), −sin(θ), 0].

The X-ray scattering length in the unrotated channel of polarization σ → σ′ is modelled by (σ′σ)/*D*(*E*). In this instance, the resonant denominator is replaced by a sharp oscillator *D*(*E*) = {[*E* − Δ + *i*Γ/2]/Δ} with the X-ray energy *E* in the near vicinity of an atomic resonance Δ of total width Γ, namely, *E* ≃ Δ and Γ << Δ. The cited energy-integrated scattering amplitude (σ′σ), one of four amplitudes, is studied using standard tools and methods from atomic physics and crystallography. In the first place, a vast spectrum of virtual intermediate states makes the X-ray scattering length extremely complicated. It can be truncated by closely following the steps in celebrated studies by Judd and Ofelt of optical absorption intensities of rare-earth ions (Judd, 1962 ▸; Ofelt, 1962 ▸; Hehlen *et al.*, 2013 ▸). An intermediate level of truncation used here reproduces sum rules for axial dichroic signals created by *E*1–*E*1 or *E*2–*E*2 absorption events (Johnson & Lovesey, 2024 ▸). The attendant calculation presented in Lovesey & Balcar (1997 ▸) and Section 5.2 in Lovesey *et al.* (2005 ▸) is lengthy and demanding. Experimental results for Dirac multipoles in V_2_O_3_ (Fernández-Rodríguez *et al.*, 2010 ▸) and CuO (Scagnoli *et al.*, 2011 ▸; Lovesey & Balcar, 2013 ▸) have been published together with successful interpretations. The study of V_2_O_3_ is noted its full use of linear photon polarization analysis to disentangle multipoles in diffraction amplitudes.

Here, we implement universal expressions for scattering amplitudes and abbreviate notation using (σ′σ) ≡ *F*_σ′σ_*etc*. for *E*1–*E*1 amplitudes listed by Scagnoli & Lovesey (2009 ▸, Appendix C); likewise, *E*1–*E*2 amplitudes by Scagnoli & Lovesey (2009 ▸, Appendix D) and *E*1–*M*1 amplitudes, where *M*1 is the magnetic dipole moment (Lovesey & Balcar, 2010*a* ▸; Lovesey & Balcar, 2010*b* ▸).

Laue conditions for magnetic reflections (0, 1, 0) and (0, 0, 1) are satisfied at the nickel *L*_3_ and *L*_2_ absorption edges at *E* ≃ 0.861 keV and *E* ≃ 0.878 keV, respectively. The relevant unit-cell parameters for Li_2_Ni_3_P_4_O_14_ are *b* ≃ 7.749 Å and *c* ≃ 9.337 Å (Chikara *et al.*, 2025 ▸), *e.g.* sin(θ) = [λ/{2*c*sin(β_o_)}] for (0, 0, 1) with a photon wavelength λ ≃ (12.4/*E*) Å. An *E*1 (2*p* → 3*d*) event is much stronger than *E*1 (1*s* → 4*p*) and *E*2 (1*s* → 3*d*) events at the nickel *K* edge at *E* ≃ 8.339 keV (Paolasini, 2014 ▸).

## 4. **Reflections** (0, 2*n* + 1, 0)

Reflections (0, 2*n* + 1, 0) violate the condition for Thomson and nuclear scattering by ions at positions 2*c* and 4*e*. The crystal axis *c* is normal to the plane of scattering depicted in Fig. 2 ▸. There is no *E*1–*E*1 diffraction in the unrotated σ-channel, *i.e.* (σ′σ) = 0. This null result follows from two facts [Scagnoli & Lovesey (2009 ▸), Appendix C]: a well known absence of contributions to (σ′σ) from magnetic dipoles, and all quadrupoles allowed by Neumann’s principle are invisible in reflections (0, 2*n* + 1, 0). The first fact is not valid for *E*2–*E*2 diffraction, and magnetic dipoles 〈*T*^1^_ζ_〉 and 〈*T*^1^_ξ_〉 contribute in (σ′σ) [Scagnoli & Lovesey (2009 ▸), Appendix E]. Two remaining (0, 2*n* + 1, 0) *E*1–*E*1 diffraction amplitudes are
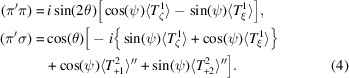
A factor {2 cos(2π*ky*)} with *y* ≈ 0.1314 (Chikara *et al.*, 2025 ▸) is omitted in equation (4) for Wyckoff positions 4*e*, and it is returned in equation (6). The rotated channel amplitude (π′σ) demonstrates that contributions from axial magnetic dipoles and non-magnetic Templeton–Templeton (T&T) scattering are shifted in phase by 90° (Templeton & Templeton, 1985 ▸; Templeton & Templeton, 1986 ▸; Ovchinnikova *et al.*, 2025 ▸). Scattered intensity picked out by circular polarization in the primary photon beam equals *P*_2_ϒ with a chiral signature (Tanaka *et al.*, 2010 ▸)
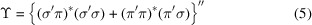
and the Stokes parameter *P*_2_ (a purely real pseudoscalar) measures helicity in the primary X-ray beam. Since intensity is a true scalar, ϒ and *P*_2_ must possess identical discrete symmetries, specifically, both scalars are time-even and parity-odd. The signature is extracted from observed intensities by subtraction of intensities measured with opposite handed primary X-rays, namely, ±*P*_2_. Intensity of a Bragg spot in the rotated channel of polarization is proportional to |(π′σ)|^2^, and likewise for unrotated channels of polarization. The corresponding *E*1–*E*1 chiral signature is
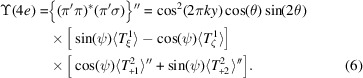
Notably, the chiral signature is a product of dipoles and T&T scattering, and a function of sin(2ψ) and cos(2ψ). The *E*2–*E*2 chiral signature is much more complicated because (σ′σ) ≠ 0.

An *E*1–*E*2 absorption event reveals Dirac multipoles 〈**G**^*K*^〉 with *K* = 1, 2, 3 hosted only by Wyckoff positions 4*e* [Scagnoli & Lovesey (2009 ▸), Appendix D:2]. We consider the unrotated amplitude (σ′σ) in light of the absence of diffraction in this channel for an *E*1–*E*1 event. The Dirac amplitude is purely imaginary and proportional to {sin(2π*ky*)cos(θ)}. Working to the level of the diagonal octupole
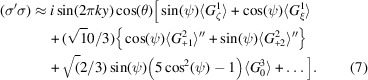
The three omitted octupoles are 〈*G*^3^_*Q*_〉′ with *Q* = 1–3. Anapoles (*K* = 1) in (σ′σ) are parallel to a* and **c**. The four amplitudes have identical phases, namely, purely imaginary, and the *E*1–*E*2 chiral signature is zero.

## 5. **Reflections** (0, 0, 2*n* + 1)

Diffraction amplitudes for space group forbidden reflections (0, 0, 2*n* + 1) depend on the monoclinic obtuse angle, and we use *d* = cos(β_o_) and *e* = sin(β_o_) for an *E*1–*E*1 absorption event. It is convenient to employ multipoles
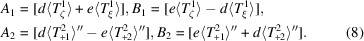
Axial dipoles in *A*_1_ and *B*_1_ are parallel to a* and **c**, and T&T scattering create *A*_2_ and *B*_2_.

The cited amplitudes possess common factors {4cos(2π*lz*)} with *z* ≈ 0.9789 (Chikara *et al.*, 2025 ▸) or 2 for Wyckoff positions 4*e* and 2*c*, respectively. The unique axis **b** is parallel to the axis *y* in Fig. 2 ▸ at the start of an azimuthal angle scan ψ = 0.*E*1–*E*1 amplitudes are
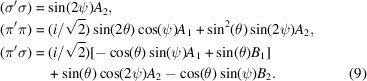
The unrotated amplitude (σ′σ) is purely non-magnetic T&T scattering. Absence of the Bragg angle θ in (σ′σ) is a general condition [Scagnoli & Lovesey (2009 ▸), Appendix C]. Multipoles *A*_1_ and *A*_2_ in the amplitudes (π′σ) and (σ′π) take opposite signs. The chiral signature is caused by an interference between axial dipoles and T&T scattering
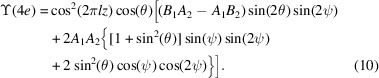
The expression ϒ(4*e*) is a sum of odd and even functions of the azimuthal angle.

An *E*1–*E*2 absorption event reveals the anapole parallel to the unique axis **b** in the unrotated amplitude (σ′σ),
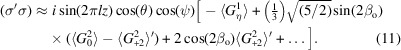
Octupoles omitted in equation (11) are 〈*G*^3^_*Q*_〉′′ with *Q* = 1–3. In line with reflections (0, 2*n*+ 1, 0), the four amplitudes have identical phases and the *E*1–*E*2 chiral signature is zero.

## 6. Summary and discussion

We have studied Bragg diffraction patterns for the low-temperature phase of Li_2_Ni_3_P_4_O_14_. On the basis of an extensive set of investigations, Chikara *et al.* (2025 ▸) conclude that it is a monoclinic, collinear antiferromagnet with nickel ions using two Wyckoff positions in the magnetic space group *P*2_1_/*c* (No. 14.75) depicted in Fig. 1 ▸. Symmetry-informed calculations of resonant X-ray Bragg diffraction patterns reported in the main text include Dirac multipoles (polar and magnetic) permitted at the position that is asymmetric. Anapoles (Dirac dipoles) engaged in diffraction are parallel to the unique axis **b**, and a* and **c**, respectively, for two classes of space group forbidden reflections. The magnetic crystal class (2/*m*) permits the coupling of the magnetic structure to circular polarization (helicity) in the primary beam of X-rays using a parity-even electric-dipole electric-dipole (*E*1–*E*1) absorption event. In this study, the coupling is quantified by a chiral signature proportional to the change in X-ray helicity on scattering (Tanaka *et al.*, 2010 ▸). The chiral signature is zero for Dirac multipoles exposed by a parity-odd electric-dipole electric-quadrupole (*E*1–*E*2) absorption event. Calculated diffraction patterns are enriched by simulating azimuthal angle scans in which the crystal is rotated about the reflection vector.

Chikara *et al.* (2025 ▸) did not allow Dirac multipoles in the analysis of their magnetic Bragg diffraction patterns gathered with neutron scattering (Lovesey *et al.*, 2019 ▸). The technique measures the spatial distribution of magnetization (Brown, 1993 ▸). Their published measurements extend to a small *d*-spacing (wavevector κ ≈ 1.36 Å^−1^) that spans a maximum in the radial integral that accompanies an anapole and a Dirac quadrupole (Lovesey, 2015 ▸; Lovesey & van der Laan, 2024 ▸).
